# A Case of Sigmoid Colon Perforation Associated with Sodium Zirconium Cyclosilicate in a Patient with Advanced Rectal Cancer

**DOI:** 10.70352/scrj.cr.24-0123

**Published:** 2025-03-11

**Authors:** Takaki Kamiya, Toru Miyake, Osamu Inatomi, Tomoharu Shimizu

**Affiliations:** 1 Department of Pharmacy, Shiga University of Medical Science Hospital, Otsu, Shiga, Japan; 2 Medical Safety Section, Shiga University of Medical Science Hospital, Otsu, Shiga, Japan; 3 Department of Surgery, Shiga University of Medical Science, Otsu, Shiga, Japan; 4 Department of Gastroenterology, Shiga University of Medical Science, Otsu, Shiga, Japan

**Keywords:** sodium zirconium cyclosilicate, hypokalemia, sigmoid colon perforation, drug-induced gastrointestinal disorders

## Abstract

**INTRODUCTION:**

Sodium zirconium cyclosilicate (SZC) binds with potassium in the gastrointestinal tract and increases fecal potassium excretion. Since SZC is a novel and non-swelling drug, the risk of intestinal perforation is not mentioned in the package insert. We hereby report the case of sigmoid colon perforation caused by hard stools and severe hypokalemia in a patient suffering from advanced rectal cancer, who was taking SZC.

**CASE PRESENTATION:**

A woman in her 80s with a history of primary biliary cirrhosis and decompensated cirrhosis accompanied by hyperkalemia was administered SZC. The patient was rushed to the hospital on the 23rd day following the commencement of SZC, complaining of abdominal pain and nausea. She suffered from sigmoid colon perforation. Hartmann’s operation with drainage was performed. SZC was discontinued after admission, following which the serum potassium levels normalized. Despite the diagnosis of advanced rectal cancer during her hospital stay, the curative operation and stoma closure were judged to be inoperable because of her physical condition.

**CONCLUSIONS:**

SZC is said to be associated with a lower risk of intestinal perforation compared with other similar drugs; however, in patients with certain conditions, such as intestinal obstruction or transit disorder resulting from malignant disease, adhesion, and inflammatory diseases, the accumulation of SZC in the intestinal lumen might increase the risk of perforation.

## Abbreviations

AKI
: acute kidney injury
CPS
: calcium polystyrene sulfate
CT
: computed tomography
HD
: hemodialysis
HU
: Hounsfield units
ICU
: intensive care unit
SPS
: sodium polystyrene sulfate
SZC
: sodium zirconium cyclosilicate

## INTRODUCTION

Sodium zirconium cyclosilicate (SZC) is a highly selective cation exchanger that traps potassium in the intestinal tract in exchange for sodium and hydrogen.^1–3)^ SZC binds with potassium in the lumen of the gastrointestinal tract, thereby reducing the concentration of free potassium in the gastrointestinal lumen, consequently lowering serum potassium levels and increasing fecal potassium excretion.^4)^ Special warnings and precautions associated with potassium binders other than SZC include hypokalemia, QT prolongation, the risk of interaction with X-rays, and intestinal perforation. To date, no events of intestinal perforation have been reported as being caused by SZC,^5,6)^ even though intestinal perforation has been reported with other potassium binders as well.^7–9)^ We hereby report a case of sigmoid colon perforation that accidentally occurred when the patient who had been diagnosed with advanced rectal cancer was taking SZC.

## CASE PRESENTATION

The patient was a woman in her 80s who had previously been diagnosed with primary biliary cirrhosis and decompensated cirrhosis, classified as class B (Child–Pugh score was 9). At her outpatient visit, which was 23 days prior to her surgery, her serum potassium levels were 6.1 mEq/L; however, there was no evidence of abnormalities on her electrocardiogram. She was prescribed SZC at an initial dosage of 10 g three times a day for 3 days, followed by 5 g three times a day for 25 days to treat her hyperkalemia. Telmisartan 20 mg tablet was discontinued. The patient began to take SZC as instructed after receiving the medication on the same day. The concomitant medications she was receiving were: vonoprazan 10 mg, azosemide 30 mg, tolvaptan 7.5 mg, ursodeoxycholic acid 300 mg, polaprezinc 75 mg, LIVACT granules (L-isoleucine, L-leucine, and L-valine granules), and Aminoleban EN powder. However, we could not pay attention to the safety management regarding the changes in potassium levels associated with the consumption of SZC.

The ammonia level of the patient was 24 µg/dL, which was within the normal range, approximately 1 year before admission. Furthermore, there were no problems with communication during subsequent outpatient visits, because of which the ammonia level was not measured. At the time of admission, her ammonia level was 37 µg/dL. During the course of hospitalization, her ammonia levels were measured several times and ranged between 31 and 45 µg/dL, with no evidence of abnormalities. She had been taking her medication as prescribed until her hospitalization. The patient was assisted by family members and regularly took the SZC for approximately 23 days.

**Fig. 1:**
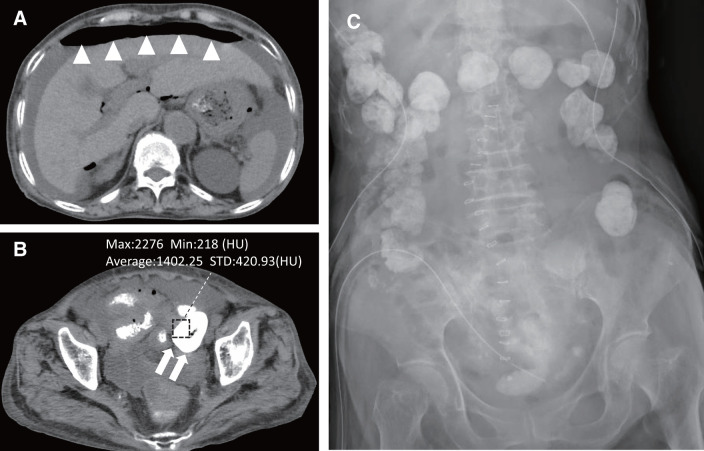
CT images at the time of admission and postoperative X-ray. Presence of massive free air patches (white triangles) in the upper abdominal area (**A**) and high-intensity feces in the extraintestinal lumen (white arrows) in the pelvic cavity (**B**) were revealed on abdominal CT imaging with HU. The postoperative X-ray image revealed a massive accumulation of SCZ in the feces (**C**). CT, computed tomography; HU, Hounsfield units; SCZ, sodium zirconium cyclosilicate

**Fig. 2:**
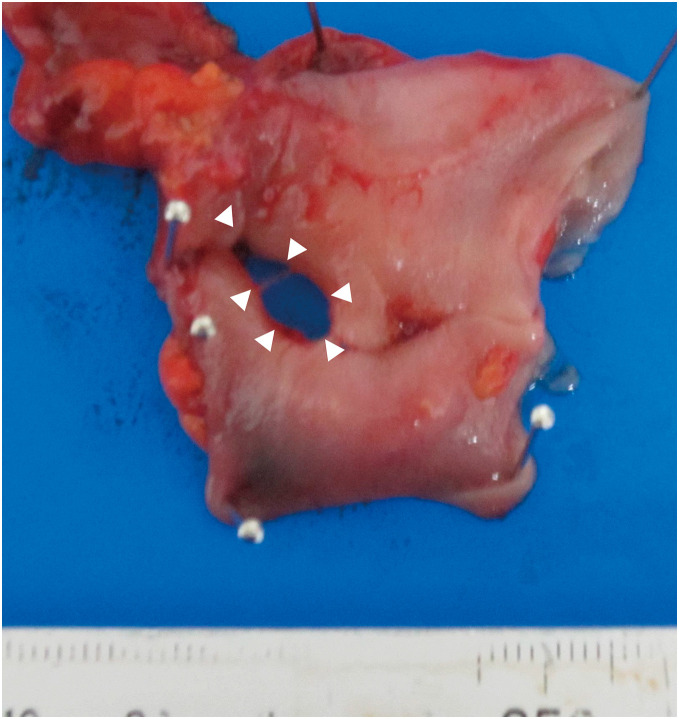
Resection specimen. The perforation size in the sigmoid colon was 13.0 mm × 7.0 mm. The area enclosed by the white triangles represents the perforation site.

The day before surgery, after having dinner at around 7 to 8 p.m., the patient experienced abdominal pain and nausea at around 10:30 p.m., and she had to be rushed to our hospital. A computed tomography (CT) scan was performed, and an axial view of the scan revealed free intraperitoneal gas and ascites in the upper abdominal space (**Fig. 1A**). A high-intensity fecal mass was identified inside the colon and outside the intestinal tract at the pelvic level (**Fig. 1B**). Subsequent laboratory analysis identified several irregularities, including a potassium level of 1.9 mEq/L, blood urea nitrogen level of 27.5 mg/dL, creatinine of 1.22 mg/dL, hemoglobin level of 8.8 g/dL, and white blood cell count of 8100/µL. However, the C-reactive protein level was 0.20 mg/dL. The estimated glomerular filtration rate was 32.0 mL/min/1.73m^2^. Emergency surgery was performed the day following her admission (defined as Day 0). Several serous ascites and free intraperitoneal gas were observed in the epigastrium due to cirrhosis. Ascites-like watery stools were observed from the left lower abdominal quadrant to the pouch of Douglas, and hard stools were observed outside the intestinal tract in the lower abdominal quadrant. Hartmann’s operation with drainage was performed for repairing the sigmoid colon perforation. The postoperative X-ray images revealed accumulation of SZC in the feces (**Fig. 1C**). The perforation size of the resected specimen was 13.0 mm × 7.0 mm, and macroscopic findings did not reveal necrosis or bleeding (**Fig. 2**). Microscopic findings did not reveal significant inflammatory cell infiltration, ischemic changes, or neoplasia around the perforation. The patient continued to receive critical care services in the intensive care unit (ICU) after surgery.

**Fig. 3:**
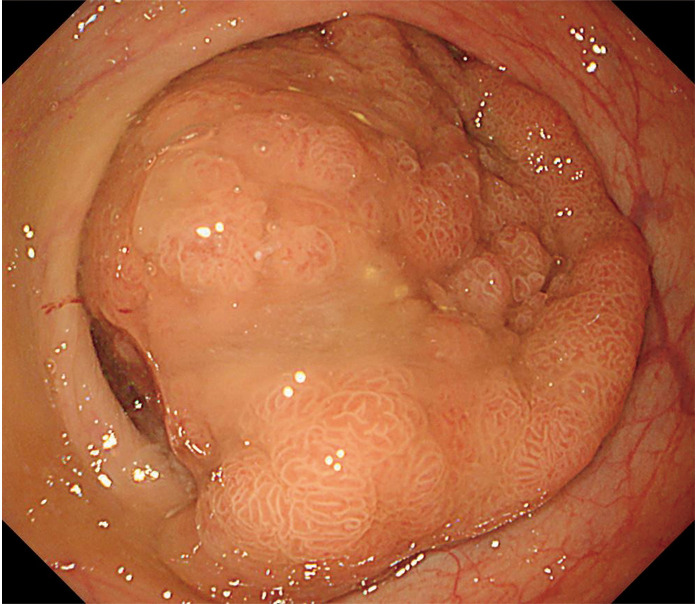
Rectal endoscopy image. A rectal tumor was observed to occupy the entire circumference of the rectal lumen.

Continuous hemodiafiltration was initiated on Day 0 due to a diagnosis of acute kidney injury (AKI), which was changed to hemodialysis (HD) on Day 7. SZC was discontinued after admission, and potassium was administered intravenously as needed. Despite the administration of multiple gastroprokinetic agents and laxatives, it took about 1 week for the hard stools containing residual SZC to be excreted. The X-ray images indicated the excretion of the residual SZC. Enteral nutrition was resumed on Day 7, and swallowing training was initiated on Day 8 following the patient’s transfer from the ICU to the medical ward. On Day 13, she developed a lacunar infarction and was treated conservatively. Her renal function improved, and HD was discontinued on Day 36. Then, on Day 51, she developed sudden circulatory failure. Cardiac catheterization revealed acute myocardial infarction caused by the obstruction of the right coronary artery. Thrombectomy and transcatheter stent placement were performed. The patient was also diagnosed with advanced rectal cancer during the hospital stay (**Fig. 3**). Curative operation and stoma closure were considered inoperable because of her physical condition.

On the 95th day following surgery, despite an improvement in her condition, it was determined that a discharge to home would be difficult; hence, the patient was transferred to another hospital for rehabilitation.

## DISCUSSION

We experienced a case of sigmoid colon perforation caused by hard stools and severe hypokalemia that accidentally resulted following the intake of SZC by a patient suffering from advanced rectal cancer. SZC is an insoluble, inorganic, non-polymer zirconium silicate compound comprising units of oxygen-linked zirconium and silicon atoms in the form of a microporous cubic lattice framework.^4)^ The package insert of SZC in Japan, similar to those in the United States and Europe, advises the monitoring of serum potassium levels at appropriate time intervals.^5,6)^ In this case, the total daily dose was not exceeded as indicated in the package insert; however, the daily dose was administered in the form of 3 divided doses. The drug was administered in 3 divided doses in a Phase 3 trial in Japan^10)^; thus, the administration of SZC was considered to have been within an acceptable range. However, even though we should have monitored her closely, serum potassium levels were not measured after the initiation of SZC administration. The patient accidentally developed severe hypokalemia, with her potassium levels dropping to 1.9 mEq/L (Common Terminology Criteria for Adverse Events, Version 5, Grade 4) at the time of hospitalization. Extreme changes in potassium levels can adversely affect gastrointestinal motility. Hypokalemia can also cause paralytic ileus. Although serum potassium levels recovered and reached the normal range following about 3 days of postoperative multidisciplinary treatment, it is possible that severe hypokalemia could have further exacerbated the sigmoid colon perforation. This patient suffered from renal dysfunction and cirrhosis, both of which could have caused brain and heart infarctions. However, the relationship between these underlying diseases and the colon perforation needs to be further discussed. Colon perforation has previously been reported in patients undergoing HD; however, the reports specific to patients with chronic kidney disease are limited.^11)^ There are some similarities to previous reports in chronic constipation and intestinal fragility of this patient, who is not undergoing HD. Considering the edema caused by cirrhosis, the fluid restriction could also have contributed to her chronic constipation. Although the underlying disease was not a direct cause, this patient might have been at high risk for developing gastrointestinal perforation.

In this case, a patient using SZC developed sigmoid colon perforation despite the absence of a clear warning of intestinal perforation on the package insert. Kosiborod *et al*., who evaluated the safety and efficacy of SZC in outpatients, reported a higher incidence of edema and hypokalemia in the high-dose group, although the difference in the incidence of constipation as an adverse event between the SZC and placebo groups was reported to be only 2% in the 10g groups.^12)^ However, the incidence of constipation of 15.3% reported in the Phase 3 trial in Japan was higher than the 9.1% reported in the HARMONIZE-Global trial.^10,13)^ Therefore, constipation should be considered as a frequent adverse effect of SZC. Our patient was prone to constipation due to the comorbidity of advanced rectal cancer and colonic transit disorders. Therefore, accumulation of hard stools in the colon after intake of SZC could be one of the risk factors for sigmoid colon perforation in the current case. This case also suggested that hard fecal impaction and several colonic diverticula could be associated with the perforation. Furthermore, the long-term use of diuretics as a solution for fluid retention associated with cirrhosis could have delayed the excretion of hard stool along with SZC.

Despite suffering from defecation disorder and taking laxatives over the past 5 years, this patient did not display any characteristic symptoms of advanced rectal cancer, such as melena, tenesmus, and narrow stools. The low hemoglobin level was almost the same within the recent year and considered due to decompensated cirrhosis. Therefore, advanced rectal cancer was not suspected or detected in a timely manner. SZC, compared with other potassium binders, is reportedly associated with a lower risk of gastrointestinal complications because of its chemical properties. Regular monitoring of potassium levels, as recommended in the package insert, would have helped confirm the potassium trend; however, it is highly likely that gastrointestinal perforation could not have been avoided in this case despite proper monitoring, as the patient was prone to constipation before the initiation of SZC. However, with an increase in the number of visits, the possibility of detecting a worsening abdominal condition increased, and perforation could have been avoided. It is recommended to assess the bowel habits of patients at each visit and, if necessary, refer them for a total endoscopy or specialist consultation.

On the other hand, a previously conducted study has reported that other similar drugs such as calcium polystyrene sulfonate (CPS) and sodium polystyrene sulfonate (SPS) cause intestinal mucositis, constipation, and intestinal perforation.^7–9)^ The functional mechanism is not known; however, changes in drug properties due to swelling, increasing intestinal pressure caused by constipation, and physical irritation resulting from hard stool retention are thought to be factors.^14)^ A case report examining colonic perforation caused by a CPS bezoar reported the possible benefits of SZC for patients at high risk of intestinal perforation in the future.^15)^ On the other hand, a recent article reported that SZC, CPS, and SPS were associated with similar and low risks of intestinal ischemia/thrombosis or other serious adverse gastrointestinal events.^16)^ SZC has been on the market since 2018; however, a few previously conducted studies have reported its radiopacity.^17,18)^ Kolesnik et al. reported that SZC, even when used appropriately as prescribed, remains in the body and may interfere with CT imaging.^17)^ In this case, a clear high-density material was observed on CT imaging, as depicted in **Fig. 1C**, which confirmed that SZC had accumulated in the form of feces in the intestinal tract due to the obstruction caused by rectal cancer. As shown in **Fig. 1B**, the density of the material was significantly higher than that of the surrounding intestinal content, resembling oral contrast material, with an average density of 1402.25 HU, which was similar to the previously reported average density of 1130 HU^17)^ and mean density of 1270 HU.^18)^ Although the value may vary depending on the duration of medication and the time of measurement, the fecal mass observed was likely to have contained the SZC. Therefore, intestinal perforation caused by SZC may not be extremely rare when compared with that caused by both CPS and SPS. Consequently, monitoring the accumulation of SZC using X-rays may lead to the early detection of intestinal adverse events during SZC administration in cases of severe constipation, thereby differentiating high-risk patients with intestinal perforation. In addition, considering the risk of increasing intestinal pressure, we should be cautious regarding the accumulation of drugs, regardless of whether the drug swells or not. In particular, if there is a possibility of gastrointestinal diseases, patients need to be made aware of the risk of constipation at the time of initiation of SZC treatment.

## CONCLUSIONS

We have reported a case of sigmoid colon perforation that was accidentally caused by hard stool impaction in a patient who was taking SZC. The patient also had a history of constipation, colonic diverticula, cirrhosis, AKI, and advanced rectal cancer; therefore, a combination of factors could have possibly induced gastrointestinal perforation resulting from increasing intestinal pressure. However, the possibility of constipation, a common side effect of SZC, cannot be ruled out and could have contributed to the sigmoid colon perforation. Although SZC is said to be associated with a lower risk of intestinal perforation compared with CPS and SPS, certain patient characteristics, such as gastrointestinal obstruction, malignant tumors, and hypokalemia, could increase the risk of perforation.

## ACKNOWLEDGMENTS

The authors gratefully acknowledge the valuable cooperation of Shin-ya Morita (Department of Pharmacy, Shiga University of Medical Science Hospital, Otsu, Japan).

## DECLARATIONS

### Funding

Not applicable.

### Authors' contributions

All authors contributed to the study’s conception and design.

TM performed the surgery.

TK and TS collected the data.

TK wrote the first draft of the manuscript.

OI and TS supervised the manuscript writing.

All authors have read and approved the final manuscript.

### Availability of data and materials

The data are not available for public access because of patient privacy concerns but are available from the corresponding author upon reasonable request.

### Ethics approval and consent to participate

Not applicable.

### Consent for publication

Informed consent was obtained from the patient for the publication of this case report and the accompanying images.

### Competing interests

The authors declare that they have no competing interests.
